# Causal Variation in Yeast Sporulation Tends to Reside in a Pathway Bottleneck

**DOI:** 10.1371/journal.pgen.1004634

**Published:** 2014-09-11

**Authors:** Kim Lorenz, Barak A. Cohen

**Affiliations:** 1 Department of Genetics and Center for Genome Sciences and Systems Biology, Washington University School of Medicine, St. Louis, Missouri, United States of America; Georgia Institute of Technology, United States of America

## Abstract

There has been extensive debate over whether certain classes of genes are more likely than others to contain the causal variants responsible for phenotypic differences in complex traits between individuals. One hypothesis states that input/output genes positioned in signal transduction bottlenecks are more likely than other genes to contain causal natural variation. The *IME1* gene resides at such a signaling bottleneck in the yeast sporulation pathway, suggesting that it may be more likely to contain causal variation than other genes in the sporulation pathway. Through crosses between natural isolates of yeast, we demonstrate that the specific causal nucleotides responsible for differences in sporulation efficiencies reside not only in *IME1* but also in the genes that surround *IME1* in the signaling pathway, including *RME1*, *RSF1*, *RIM15*, and *RIM101*. Our results support the hypothesis that genes at the critical decision making points in signaling cascades will be enriched for causal variants responsible for phenotypic differences.

## Author Summary

Distinguishing the small number of genetic variants that impact phenotypes from the huge number of innocuous variants within an individual's genome is a difficult problem. Several hypotheses concerning the location of causal variants have been put forward based on the fact that genes are often organized into signaling cascades where the activation of a gene at the top of a pathway in turn activates large numbers of downstream genes. One hypothesis states that causal variations are more likely to reside in the genes at the top of these pathways because their effects are amplified by the signaling cascade. Here we provide support for this hypothesis by showing that causal genetic variants in yeast sporulation cluster around a gene at the top of the sporulation signaling cascade. Our result suggests a way to focus the search for causal genetic variants, including those that cause disease, on a smaller number of genes that are more likely to harbor important variations.

## Introduction

Understanding the genetic architecture of complex traits is a major challenge in quantitative genetics. This includes determining what types of genes and causal variants underlie quantitative trait loci (QTL), as well as how variants interact with each other. Whether causal variants share characteristics is a topic of great debate in the quantitative genetics community. These characteristics include whether variants are located in coding or non-coding genomic regions [1], [2], in specific hotspot genes or spread throughout the genome [3], [4], and whether certain classes of genes are more likely to harbor hotspots than others [5]–[7]. One class of genes which has been suggested as natural harbors for causal variation are ‘input/output’ genes that sit at regulatory bottlenecks in genetic pathways [4], [8].

Input/output genes “integrate an extensive array of inputs, the regulatory state, and they produce an on or off transcriptional output” whose expression drives differentiation of a specific cell fate [3]. This gives the signaling pathway a characteristic hourglass shape. Many developmental pathways have this structure, with the canonical examples being trichome and bristle development in *Drosophila melanogaster*, with *shavenbaby* and *scute* as the respective input/output genes in each pathway [9], [10]. Input/output genes are more likely to be essential to organism survival, indicating they play important roles in developmental pathways [11], [12]. Location in the genetic pathway is thought to be crucial in determining the effect of variation on phenotype; if a gene affects too many traits, negative pleiotropy may select against the accumulation of causal variants [13]. On the other hand, a gene that resides towards the end of a signaling cascade may tolerate more variation, but will also affect many fewer aspects of the output response and so be less likely to have a strong effect on the phenotype. Input/output genes are positioned centrally and as such are hypothesized to be likely locations for the accumulation of causal variation, as their variants should provide strong, but specific effects on the traits they regulate.

Another pathway which conforms to this hourglass shape is the *Saccharomyces cerevisiae* sporulation signaling pathway, with the transcription factor *IME1* acting as the input/output gene [14], [15]. Sporulation includes both meiosis and spore formation, and over 300 genes have been found to be essential to the process of sporulation in laboratory strains [16]. The expression of *IME1* is influenced by many factors, including ploidy, cell cycle status, nutritional environment, respiration, and pH [14], [17]–[20]. These multiple inputs form the top bulb of the hourglass. These signals all funnel into *IME1*, and once *IME1* is expressed, it activates expression of a cascade of genes that irreversibly initiates sporulation [15]. The initiation of sporulation causes changes in the expression levels of more than 1000 genes [21], which form the bottom bulb of the hourglass.

If input/output genes harbor causal natural variation, we would expect *IME1* to contain quantitative trait nucleotides (QTNs) causing differences in sporulation. Our previous study identified four natural causal variants responsible for 80% of the difference in sporulation efficiency between a high sporulating oak tree isolate and a low sporulating vineyard strain: two in *IME1*; one in *RME1*, which directly binds the *IME1* promoter in response to ploidy; and one in *RSF1*, which regulates respiration, a process essential for sporulation [22]. These results support the hypothesis that genes surrounding the *IME1* signaling bottleneck contain causal natural variation, in addition to the input/output gene itself. However, these are results from a single pair of strains chosen to have the most extreme phenotypic differences, and it is possible that natural variation in strains with more moderate phenotypes resides in genes outside of the sporulation bottleneck. Other efforts to identify the genetic basis of differences in sporulation efficiency among yeast strains have used the laboratory strains SK1 and S288c, and have identified several genes outside of the sporulation pathway [23], [24]. However, since these two strains have been propagated in the lab for thousands of generations, and since two of the genes identified in these studies have highly pleiotropic effects [25]–[29], we do not expect the variation found in the S288c×SK1 cross to be representative of naturally occurring variation.

A meta-analysis of causal variation found that input/output genes were more likely to be hotspots, but was based on a single QTL identified per trait [7]. It is possible that the strongest QTL affecting a trait is located in the input/output gene and other QTL are more evenly distributed across other parts of the pathway. To test the hypothesis that genes surrounding the sporulation pathway bottleneck are more likely to contain natural causal variation, we performed crosses with two additional vineyard isolates to identify additional causal variants responsible for differences in sporulation efficiency. We again identified *RME1* and *IME1* as repositories of causal variation, but in addition we found two more genes that contain causal natural variation for sporulation efficiency: *RIM101* and *RIM15*. Both genes are upstream regulators of *IME1*. Our finding that the causal variation underlying differences in sporulation efficiency among vineyard isolates is clustered in genes that regulate *IME1* supports the hypothesis that bottlenecks in regulatory pathways are likely repositories of causal natural variation.

## Results

Previously we identified 4 QTN in 3 genes, *IME1*, *RME1*, and *RSF1*, which explain 80% of the difference in sporulation efficiency between YPS606, an oak tree isolate and BC187, a vineyard isolate. As these genes reside at the bottleneck in the sporulation pathway, we asked whether causal variants in other strains would also reside in bottleneck genes. In this study, we identify QTL responsible for approximately two thirds of the variation in sporulation efficiency between two new vineyard isolates, UCD51 and M5, and the same oak isolate, YPS606. We chose to use the same oak strain as in previous studies because we had previously shown very limited variation in sporulation efficiency among oak isolates [30]. The vineyard strains were chosen because while they contain the causal *RME1* vineyard variant, as all vineyard strains do, they do not contain any of the other three identified causal SNPs [22]. Though vineyard strains have been shown to have similar levels of genomic diversity compared to the oak populations [31], [32], they show differences in the sporulation phenotype. While YPS606 sporulates at 99% [30], UCD51 sporulates at 25.8% and M5 sporulates at 35.2%. We sought to identify polymorphisms that contribute to low sporulation efficiency in these two vineyard strains.

**Figure 1:**
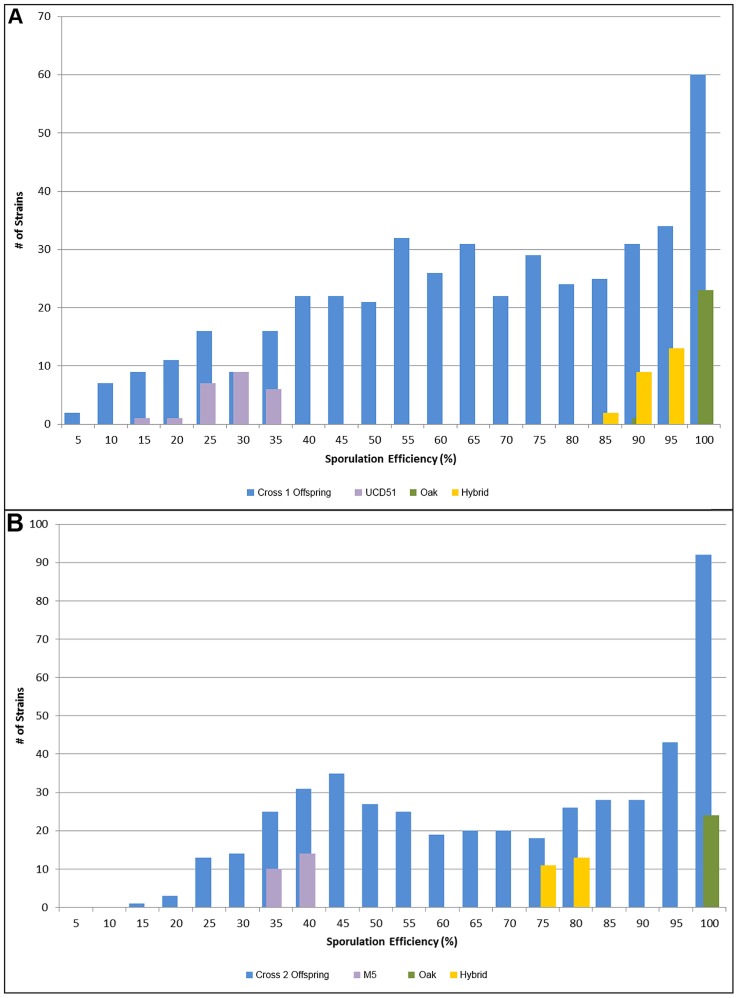
Histograms of sporulation efficiencies for progeny. In each graph, the offspring are shown in blue, with 24 replicates each of the oak parent in green, the vineyard parent in purple, and the hybrid in yellow for comparison. Number of isolates is on the y-axis, while sporulation efficiency is along the x-axis in bins of 5 percent, where 5 indicates the bin containing all isolates with sporulation efficiencies from 0%–5%, 10 contains 5%–10%, etc. A) Offspring from cross 1. Average sporulation efficiency for UCD51 is 25.8%, for YPS606 it is 98.1% and for the hybrid it is 90.6%. B) Offspring from cross 2. Average sporulation efficiency for M5 is 35.2%, for YPS606 it is 99.3% and for the hybrid it is 82.6%.

Monosporic vineyard isolates of UCD51 and M5 containing the SPS2::GFP sporulation marker were independently crossed to the previously described YPS606 oak isolate. For simplicity, the UCD51×YPS606 cross will be referred to as cross 1, and the M5×YPS606 cross will be referred to as cross 2. We phenotyped 449 doubled haploid offspring for cross 1 and 468 for cross 2 for sporulation efficiency (Figure 1). From these phenotype distributions, we calculated broad sense heritabilities of *H^2^* = 95.7% for cross 1 and *H^2^* = 99.7% for cross 2, which confirm that most of the variation in these crosses is genetic in nature. We also note that both crosses exhibit transgressive segregation, with offspring sporulating both higher and lower than the parental strains, which suggests the presence of one or more transgressive alleles.

**Figure 2:**
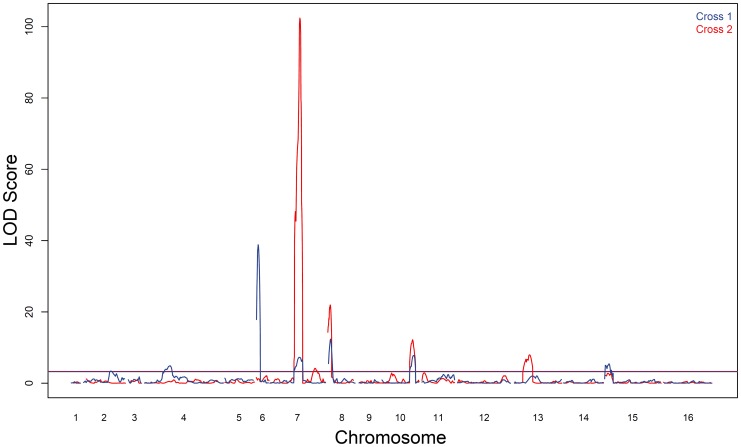
Sporulation efficiency QTL. LOD traces from cross 1 (YPS606×UCD51) are shown in blue; cross 2 (YPS606×M5) is overlaid in red. The thresholds for significance were set using 1000 permutations of each dataset; the threshold for cross 1 was 3.2 LOD while cross 2 was 3.3 LOD.

To identify QTL responsible for differences in sporulation efficiency in each cross, we used composite interval mapping (see methods). We found seven QTL in cross 1, on chromosomes 2, 4, 6, 7, 8, 10, and 15. In cross 2 we identify five QTL, two on chromosome 7, and one each on chromosomes 8, 10, and 13. LOD traces for each cross are found in Figure 2. Effect directions and genomic coordinates of the marker nearest to each QTL apex are found in Table 1.

**Table 1:** Markers nearest QTL peak apex.

Chromosome	Cross 1 Marker	Cross 2 Marker	Location	Oak Allele Effect Direction
2	M02.20	-	498594	−
4	M04.21	-	505778	+
6	M06.03	-	56274	+
7	M07.25	A07.26	558267	+
7	-	A07.39	837745	+
8	M08.02	A08.04	46929	+
10	-	A10.20	590864	−
10	M10.19	-	619165	−
13	-	A13.11	289387	+
15	M15.04	-	74696	+

Location corresponds to the start of the read mapped to the reference genome. A ‘−’ indicates that no QTL was found in at that genomic location in that cross.

As expected based on the transgressive segregation seen among the offspring of both crosses (Figure 1), there are a mixture of allele effects in each cross. The BC248 oak parent mostly contributes alleles that increase sporulation efficiency: five alleles in cross 1 (on chromosomes 4, 6, 7, 8, and 15) and four alleles in cross 2 (both QTL on chromosome 7 as well as those on chromosomes 8 and 13). The oak parent also contributes alleles in each cross that decrease sporulation efficiency (on chromosomes 2 and 10 in cross 1 and chromosome 10 in cross 2). Based on genomic position and effect direction, three QTL were shared across both crosses. The first QTL on chromosome 7 and the chromosome 8 QTL both map to the same marker in each cross while the QTL on chromosome 10 map to markers less than 30 kb apart (Table 1), suggesting these three QTL represent shared variation between these two vineyard isolates.

To determine the contribution of each QTL to variation in sporulation efficiency, we created linear models describing their effects. Using the markers nearest to each QTL apex, we applied a forward and backward stepwise regression using Bayesian information criterion (BIC) to select significant terms to create a linear model describing each cross (coefficients for cross 1 model are found in Table 2; for cross 2 model see Table 3). The model for cross 1 indicates a high amount of epistasis among QTL. All two-way interactions between the QTL on chromosomes 6, 7, and 10 contribute significantly to the model. Additionally, the interaction between the QTL on chromosomes 4 and 6 suggests the chromosome 4 QTL is entirely epistatic to the chromosome 6 QTL, as the additive term for the chromosome 4 QTL is non-significant when the two-way interaction is included in the model. The model for cross 2 identifies two two-way interactions, where the QTL on chromosome 10 interacts both with the first QTL on chromosome 7 as well as the chromosome 13 QTL. These interactions again suggest a purely epistatic QTL, where the chromosome 10 QTL acts entirely through the chromosome 7 and 13 loci. The *R* ^2^ for the cross 1 model is 0.67; for the cross 2 model it is 0.74, indicating we have captured between two thirds and three quarters of the variation in sporulation efficiency in each cross with these QTL.

**Table 2:** Cross 1 linear model coefficients using nearest marker to QTL apex.

Term	Effect	Significance	
intercept	0.90	0–0.001	
2	0.08	0–0.001	
4	−0.02	>0.05	NS
6	−0.25	0–0.001	
7	−0.22	0–0.001	
8	−0.13	0–0.001	
10	0.12	0–0.001	
15	−0.11	0–0.001	
4:6	−0.15	0–0.001	
6:7	0.12	0–0.001	
7:10	0.10	0.001–0.01	
6:10	−0.08	0.01–0.05	

**Table 3:** Cross 2 linear model coefficients using nearest marker to QTL apex.

Term	Effect	Significance	
intercept	1.00	0–0.001	
7	−0.42	0–0.001	
7B	−0.05	0–0.001	
8	−0.15	0–0.001	
10	0.01	>0.05	NS
13	−0.12	0–0.001	
7:10	0.09	0–0.001	
10:13	0.07	0.01–0.05	

To identify QTL for further analysis, we first eliminated those found in previous studies. The QTL on chromosome 7 found at 558 kb are located over the previously identified sporulation gene *RME1*, and both parental vineyard isolates contain the polymorphism previously shown to decrease sporulation [22]. The QTL regions found on chromosome 10 in both crosses and 13 in cross 2 were identified and explored in a previous study [33]. As these loci had been already been examined in detail, we chose to focus on other QTL for further analysis. The QTL located on chromosomes 2, 4, and 15 in cross 1 and on chromosome 7 at 838 kb in cross 2 have small effect sizes and large genomic intervals, so we did not attempt to identify QTG. Instead we used these intervals to confirm that SNP rates in sporulation genes were similar to those of genes not in the sporulation pathway. These QTL contain a total of 27 genes found to affect sporulation in a survey of the yeast deletion collection [16], which we compared with an equal number of genes from the same intervals that do not affect sporulation. We find the polymorphism rate in these sporulation genes is indistinguishable from that of the non-sporulation genes (Wilcoxian test, *P* = 0.67).

**Figure 3:**
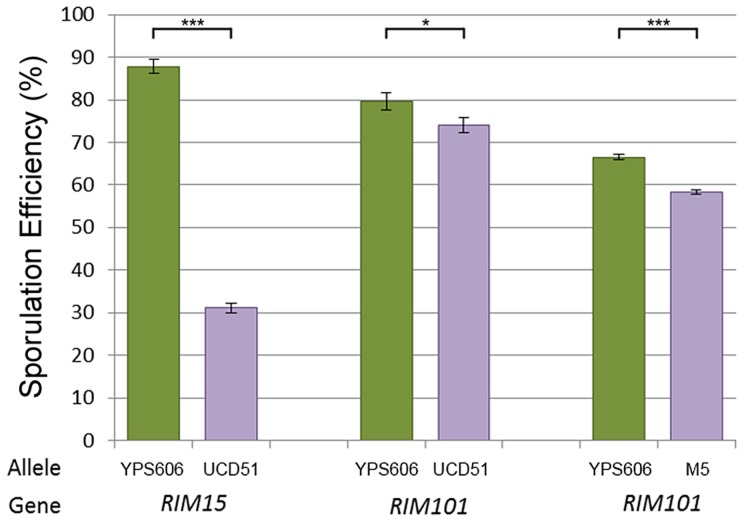
Reciprocal hemizygosity analysis. Bar color and allele label correspond to the allele present in the hemizygous strain; green – vineyard allele knockout, oak allele remaining, purple – oak allele knockout, vineyard allele remaining. Error bars show standard deviation among at least 4 independent knockouts (t-test, *** indicates *P*<0.001, * indicates *P*<0.01).

We therefore sought to identify QTG under the QTL on chromosomes 6 (cross 1) and 8 (both crosses). In addition to being in previously untested genomic regions, each of these QTL have reasonable effect sizes in our linear models and good candidate genes in their 99% confidence interval (approximated as a 2 LOD drop from the apex of the QTL). We tested candidate quantitative trait genes *RIM15* located on chromosome 6 and *RIM101* located on chromosome 8 in each QTL using reciprocal hemizygosity tests [29]. Since the QTL on chromosome 8 was identified in both crosses, both vineyard parents were used to create two sets of reciprocal hemizygotes; for *RIM15* only UCD51 was used to create reciprocal hemizygotes. Figure 3 shows the results for the three sets of reciprocal hemizygotes tested; all three show significant differences between alleles (*t*-test, *P*<0.01). As expected based on the effect directions predicted by our linear model, the hybrid strains containing the oak alleles of *RIM15* and *RIM101* have increased sporulation efficiency while the hybrid strains containing only the vineyard allele have reduced sporulation efficiency. These results indicate that *RIM15* underlies the chromosome 6 QTL in cross 1 while *RIM101* underlies the chromosome 8 QTL in both crosses.

**Figure 4:**
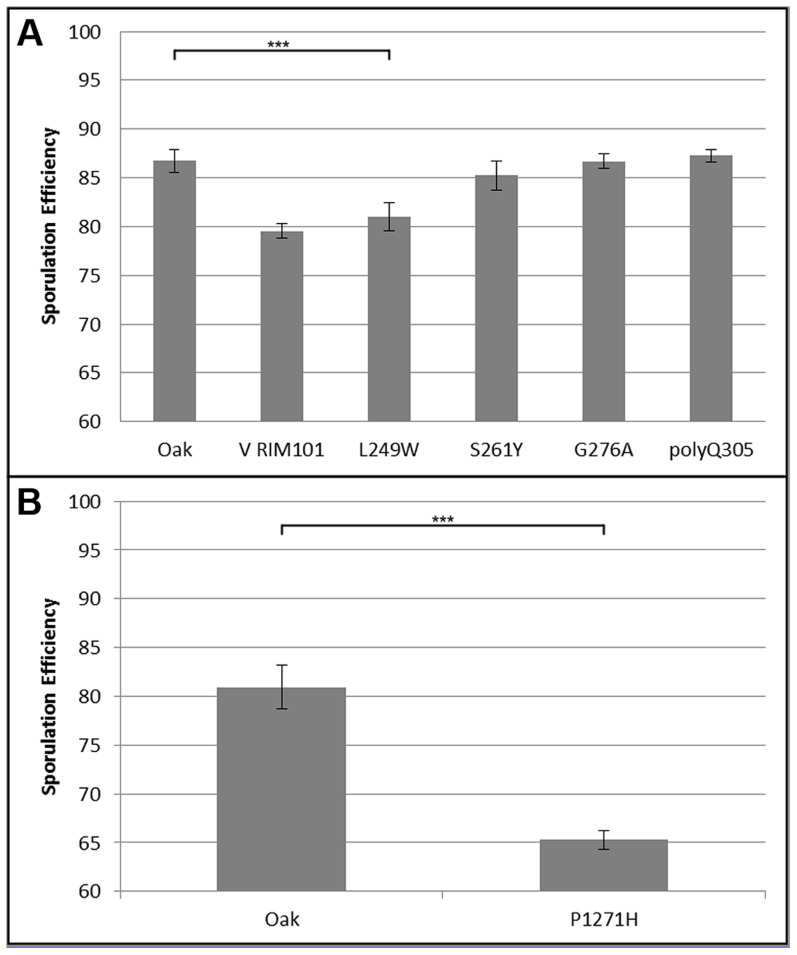
Single allele replacement analysis. All replacements were performed in the BC248 oak background. WT indicates unaltered oak strain. Error bars indicate standard deviation among at least 4 replicates (t-test, *** indicates *P*<0.001). A) *RIM101* replacements. V RIM101 indicates the full (coding and noncoding) *RIM101* gene was replaced with the M5 vineyard version; all other bars are single allele replacements as indicated. B) *RIM15* single allele replacement.

We began our search for causal polymorphisms with *RIM101*, as it was identified in both crosses. Most of the variation in *RIM101* is shared between the vineyard isolates, and there are no obvious candidate causal polymorphisms. As compared to the oak sequence, *RIM101* contains 27 SNPs and 3 insertion/deletions (indels) which are common to both UCD51 and M5. Ten of the SNPs cause non-synonymous coding changes and there is a polyQ expansion in YPS606. A conserved domain structure predictor identifies zinc finger regions covering 75% of the coding sequence [34] and encompassing most polymorphisms. Since we could not take the candidate approach to identify the causal polymorphism(s) in *RIM101*, we used a random replacement approach to identify regions of interest within the gene (see methods). Only one region showed a significant phenotypic difference between oak and vineyard alleles, (t-test, *P* = 5×10^−4^). This region contains the coding portion of *RIM101* between nucleotide positions 576 and 940, including 6 SNPs and the polyQ indel, all of which are present in both vineyard isolates used in our crosses. Two of the SNPs are synonymous and another two are in the same codon, resulting in a total of four protein differences to assay. We replaced each of these four alleles individually in the oak background and then backcrossed the single allele swap strains to the oak parent to remove any transformation induced mutations. We also backcrossed one of the complete locus replacements created during the random replacement to obtain an oak strain homozygous for the entire M5 suite of polymorphisms in *RIM101*. The phenotypes of the allele replacements tested are shown in Figure 4A. Only the G746T SNP shows a significant difference from the unaltered oak isolate (t-test, *P* = 5.5×10^−9^). This SNP results in a W249L amino acid substitution, with the oak T allele changing a tryptophan into a leucine.

We also identified a causal polymorphism in *RIM15*. Since *RIM15* was identified only in cross 1, using UCD51, and not in cross 2 or previous crosses using BC187 [22], [33], we reasoned that it was likely that the causal allele was unique to UCD51. While there are 80 SNPs and 8 indels between the vineyard UCD51 isolate and the oak YPS606 isolate, only 5 SNPs and 1 indel are not also found in BC187. *RIM15* is a glucose repressible protein kinase which regulates the formation of the IME1-UME6 complex necessary for sporulation [35], [36]. One of these SNPs, C3812A, results in a P1271H substitution and is located in a predicted kinase extension domain [34]. This proline residue is also conserved in *S. paradoxus*, *S bayanus*, *S. mikatae*, and *S. castellii*, further suggesting that this residue is a good candidate causal variant [37]. We tested this hypothesis by creating the allele replacement for C3812A in the oak background and found that its sporulation efficiency was significantly different from the unmodified oak isolate (Figure 4B, t-test, *P* = 2.6×10^−23^). Since we did not test all of the polymorphisms in *RIM15*, it is possible there are additional causal alleles in the gene.

To determine how common the identified causal SNPs are, we assayed a panel of oak and vineyard isolates for both alleles (Table 4). We found that while *RIM101* W249L segregates based on oak or vineyard classification, *RIM15* P1271H was not present in any other isolate we assayed, regardless of oak or vineyard background, including the 23 strain sequences available on the *Saccharomyces* Genome Database [37]. This suggests that the *RIM101* W249L allele was fixed early in the divergence of vineyard yeasts, while the *RIM15* P1271H variant is a more recent change that is unique to the UCD51 vineyard isolate.

**Table 4:** Causal SNP frequency in oak and vineyard isolates.

Strain	Habitat	*RIM101(W249L)*	*RIM15(P1271H)*
YPS606	Oak	T	C
UCD51	Vineyard	G	A
M5	Vineyard	G	C
BW-1	Oak	T	C
CP-1	Oak	T	C
IL-01	Oak	T	C
IN-1	Oak	T	C
NC-02	Oak	T	C
T7	Oak	T	C
TN-1	Oak	T	C
YPS142	Oak	T	C
BC187	Vineyard	G	C
M13	Vineyard	G	C
M15	Vineyard	G	C
M22	Vineyard	G	C
M29	Vineyard	G	C
M30	Vineyard	G	C
M33	Vineyard	G	C
M34	Vineyard	G	C
M7	Vineyard	G	C
RM11-1a	Vineyard	G	C
UCD175	Vineyard	G	C
UCD522	Vineyard	G	C
UCD762	Vineyard	G	C
UCD765	Vineyard	G	C
UCD781	Vineyard	G	C
UCD820	Vineyard	G	C

To investigate how our newly identified sporulation QTN interact with previously identified QTN, we crossed the *RIM101* and *RIM15* vineyard polymorphisms into a previously created YPS606 oak strain background allele replacement panel containing the *RME1*, *RSF1*, *IME1*-coding, and *IME1*-noncoding polymorphisms [22]. These panels provide sets of nearly isogenic strains containing all possible combinations of causative alleles in a constant background, and are powerful tools for assessing epistasis between causal variants. We used these new *RIM101* and *RIM15* allele replacement panels to create backwards regression linear models to identify interactions among QTN. We found that the *RIM101* QTN interacts with the both the *RME1* and *IME1*-coding QTN, but that the interaction with the *RME1* QTN is only significant when the *IME1*-coding QTN is considered (Table 5). *RIM15* on the other hand, shows extensive interactions with all previously identified QTN, again with the *RME1* interaction only contributing significantly when one of the other three QTN are taken into account (Table 6). These results provide further evidence that interactions between QTN are common, even between QTN that are not co-segregating in natural populations.

**Table 5:** *RIM101* allele replacement panel linear model.

Term	Effect	Percent Phenotypic Variance Explained	Significance	
intercept	1.01		0–0.001	
RIM101	−0.03	1.40	0–0.001	
RME1	−0.18	10.99	0–0.001	
RSF1	−0.04	1.52	0–0.001	
IME1-C	−0.18	11.10	0–0.001	
IME1-NC	−0.03	1.37	0–0.001	
RIM101:RME1	−0.01	1.07	>0.05	NS
RME1:RSF1	−0.20	7.73	0–0.001	
RIM101:IME1-C	−0.03	1.22	0–0.001	
RME1:IME1-C	−0.27	12.99	0–0.001	
RSF1:IME1-C	−0.26	12.19	0–0.001	
RME1:IME1-NC	−0.17	5.72	0–0.001	
RSF1:IME1-NC	−0.14	4.14	0–0.001	
IME1-C:IME1-NC	−0.13	3.59	0–0.001	
RIM101:RME1:IME1-C	0.06	1.31	0–0.001	
RME1:RSF1:IME1-C	0.33	9.73	0–0.001	
RME1:RSF1:IME1-NC	0.15	2.95	0–0.001	
RME1:IME1-C:IME1-NC	0.24	5.52	0–0.001	
RSF1:IME1-C:IME1-NC	0.14	2.65	0–0.001	
RME1:RSF1:IME1-C:IME1-NC	−0.13	1.76	0–0.001	
Total Phenotypic Variance Explained		98.95		

**Table 6:** *RIM15* allele replacement panel linear model.

Term	Effect	Percent Phenotypic Variance Explained	Significance	
intercept	0.99		0–0.001	
RIM15	−0.08	2.84	0–0.001	
RME1	−0.14	5.79	0–0.001	
RSF1	−0.04	1.45	0.001–0.01	
IME1-C	−0.17	8.81	0–0.001	
IME1-NC	−0.03	1.36	0.001–0.01	
RIM15:RME1	0.01	1.10	>0.05	NS
RIM15:RSF1	−0.15	3.79	0–0.001	
RME1:RSF1	−0.18	5.24	0–0.001	
RIM15:IME1-C	−0.23	7.31	0–0.001	
RME1:IME1-C	−0.27	10.19	0–0.001	
RSF1:IME1-C	−0.26	9.46	0–0.001	
RIM15:IME1-NC	−0.03	1.20	0.01–0.05	
RME1:IME1-NC	−0.17	4.65	0–0.001	
RSF1:IME1-NC	−0.12	2.85	0–0.001	
IME1-C:IME1-NC	−0.13	3.28	0–0.001	
RIM15:RME1:RSF1	0.09	1.59	0–0.001	
RIM15:RME1:IME1-C	0.22	3.96	0–0.001	
RIM15:RSF1:IME1-C	0.16	2.65	0–0.001	
RME1:RSF1:IME1-C	0.27	5.83	0–0.001	
RIM15:RME1:IME1-NC	0.09	1.58	0–0.001	
RME1:RSF1:IME1-NC	0.1	1.70	0–0.001	
RIM15:IME1-C:IME1-NC	0.1	1.66	0–0.001	
RME1:IME1-C:IME1-NC	0.22	4.33	0–0.001	
RSF1:IME1-C:IME1-NC	0.12	1.94	0–0.001	
RIM15:RME1:RSF1:IME1-C	−0.12	1.46	0–0.001	
RIM15:RME1:IME1-C:IME1-NC	−0.14	1.59	0–0.001	
RME1:RSF1:IME1-C:IME1-NC	−0.08	1.30	0.001–0.01	
Total Phenotypic Variance Explained		98.91		

## Discussion

**Figure 5:**
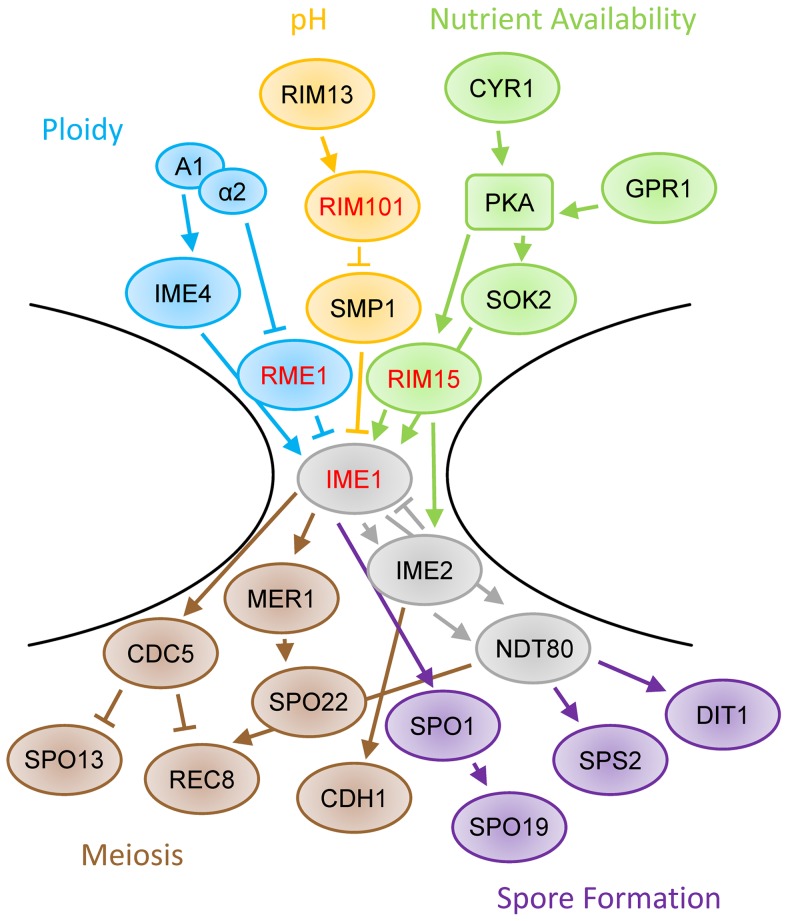
Schematic of sporulation pathway. The regulation of *IME1* involves inputs such as ploidy (blue), pH (orange), and nutrient availability (green). The induction of IME1 modifies the activity of many genes, including those involved in meiosis (brown) and spore formation (purple). Genes identified as QTL are highlighted with red text. Only a subset of sporulation-involved genes are shown. For more detail, please see [14], [15], [17], [38]–[40].

All of the quantitative trait genes for sporulation efficiency we have thus far identified in natural strains act at the bottleneck of the sporulation decision pathway at *IME1* (Figure 5; only a subset of sporulation genes shown, see [14], [15], [17], [38]–[40] for more detail). Previously, we identified three transcription factors at this bottleneck, *RME1*, *IME1*, and *RSF1* [22]. With this study we add another transcription factor, *RIM101*, and a kinase, *RIM15*. *RIM15* is responsive to glucose and helps *IME1* activate downstream sporulation genes [35], [41]. Specifically, Rim15 removes Sin3 and Rpd3 from Ume6, which allows Ime1 to bind and activate early meiotic genes [36]. *IME1* and *RIM15* work together to create the proper set of sporulation signals in response to a given nutritional environment, and the vineyard alleles in *RIM15* appear to slow that response. In this context, the genetic interactions we observe between *RIM15* and other genes in the sporulation pathway seem likely to have some basis in physical interactions between the genes involved.

*RIM101*'s contribution to the regulation of sporulation efficiency is more complex. It was initially identified as a zinc finger containing transcriptional activator of *IME1* [42], placing it upstream of the initial sporulation decision, likely through *SMP1* regulation of *IME1* [43]. Its expression and cleavage into an active form are stimulated by alkaline growth conditions, however Rim101 cleavage has also been observed prior to detectable pH increases [19], [44]. Cleaved Rim101 has been shown to regulate the mid to late sporulation genes *DIT1* and *DIT2* as well as *RIM8*, one of the early sporulation genes required for Rim101 cleavage [43], [45], [46], suggesting that *RIM101* regulates various processes throughout the sporulation signaling cascade in addition to *IME1*. Finally, cells which are *rim101*Δ cannot respond to pH based cell-cell signaling during sporulation, which normally controls efficiency and patterning in solid colonies [18], suggesting that *RIM101* may also be involved in intercellular communication. It remains to be seen what subset of these functions the W249L allele of *RIM101* affects during sporulation in vineyard and oak yeast isolates.

Overall, we identified nine different genomic regions in crosses with two new vineyard isolates that contain sporulation efficiency QTL. Of these regions, five were found in previous studies [22], [33]. We expected to find at least one QTL in common with our previous work, as the *RME1* allele we identified in BC187 is fixed across all vineyard isolates we assayed [22]. The causal alleles we identified in *RIM101* also segregate perfectly between oak and vineyard isolates, suggesting they too are fixed across these two populations. The four QTL identified in this study which had not been identified in previous crosses suggest that many variants involved in changes in sporulation efficiency are specific to individual vineyard isolates. We mapped one of these QTL to the QTG *RIM15* and identified a causal SNP. This polymorphism is present in only UCD51 and not in any of the other 48 isolates assessed. While without causal polymorphisms we cannot assess the prevalence of the other three newly identified QTL, the absence of QTL in two of the three vineyard isolates we have so far assayed suggests that they are not fixed alleles in the vineyard strains. We also confirmed that polymorphism rates are not significantly different between sporulation and non-sporulation genes in these other QTL.

In a previous study, a QTL was found over *RIM101* when the four large effect QTN were fixed as oak alleles in a cross between the same YPS606 oak strain and vineyard strain BC187. No QTL was identified in the reciprocal cross fixing the large effect alleles as the vineyard variants [33]. This indicates there is strong epistasis between one of the oak large effect QTN and what we now know to be *RIM101*. By adding the *RIM101* QTN to our allele replacement panel, we have identified significant interactions between the *RIM101* QTN, *IME1*-coding QTN, and *RME1* QTN. These interactions were enough to mask the effect of the *RIM101* QTN in the initial BC187×YPS606 cross. These sorts of epistatic masking interactions, also known as compositional epistasis, have been proposed to be at least partially responsible for the ‘missing heritability’ problem commonly encountered in human GWAS studies [47]–[49], and they do not appear to be an infrequent occurrence. We previously identified a QTL in the same region of chromosome 10 in the BC187×YPS606 fixed cross containing the large effect vineyard variants [33]. Both chromosome 10 QTL identified in this study exhibit epistatic interactions with the chromosome 7 QTL over *RME1*, suggesting that the chromosome 10 QTL effect present in all three crosses depends on the *RME1* allele present in all vineyard strains. Both the *RIM101* QTN and the chromosome 10 QTL are excellent examples of how epistatic interactions can camouflage causal variants underlying complex traits.

In this study we have identified two new QTGs: *RIM101* and *RIM15*. We determined the causal SNP in *RIM101* is a W249L substitution that is conserved among vineyard isolates, and that a causal SNP in *RIM15* that results in a P1271H substitution is unique to the UCD51 vineyard isolate. We note that all of the QTGs we identified are known sporulation genes involved in the sporulation initiation regulatory decision at *IME1*. Our results support the hypothesis that causal variation in complex traits clusters around pathway bottlenecks, not just at input/output genes.

## Materials and Methods

### Strains

Parental oak isolate BC248 was derived from YPS606 and was described previously [30]. Parental vineyard isolate UCD51 was originally collected from Burgundy, France in 1948 and is available from the Phaff yeast culture collection at the University of California, Davis. Parental vineyard isolate M5 was originally collected from an Italian vineyard in 1993; both vineyard isolates were provided to us by Justin Fay [31]. UCD51 and M5 were transformed with a GFP reporter fused to the *SPS2* ORF and marked by the *kanMX4* resistance cassette, conferring resistance to G418 [50], then sporulated to create monosporic isolates BC812 (UCD51) and BC815 (M5) containing the SPS2::GFP fusion marked by the *kanMX4* cassette. BC248 contains the *SPS2*::GFP fusion marked by the *hygMX4* cassette, which confers resistance to hygromycin [51]. BC812 (UCD51)×BC248 (YPS606) is called cross 1 throughout this manuscript, while BC815 (M5)×BC248 (YPS606) is called cross 2. For each cross, double haploid offspring were collected as tetrads. For cross 1, 476 offspring were collected, 449 were phenotyped, and 308 were genotyped. Of the cross 1 offspring genotyped, 292 had reliable phenotypes and were used for QTL mapping analysis. For cross 2, 468 offspring were collected and phenotyped; 338 of these were genotyped and used for QTL mapping.

### Genotyping and QTL Analysis

DNA was extracted using the ZR-96 Fungal/Bacterial DNA Kit (Zymo Research, Orange, CA). Markers were chosen and genotyped using a modified RAD-tag approach described previously [33]. Briefly, extracted DNA was digested using MfeI and MboI (NEB), then ligated to barcoded Illumina sequencing adapters (IDT, sequences available in table S1 of reference 33). Ligated samples were then pooled in groups of 48 (44 offspring and 2 each parental strain replicates), prepared for sequencing and sequenced using standard primers. Cross 1 pools A-G and cross 2 pools A, B, E and F were sequenced using the Illumina GAIIx platform; cross 2 pools C, D, I and J were sequenced using the Illumina HiSeq platform. Any reads longer than 36 bp were trimmed to 36 bp for analysis purposes. Raw sequencing reads for both crosses can be found in the sequence read archive (SRA) at SRP036836.

To select markers, reads were binned by barcode, barcodes were removed and reads were consolidated into unique sequences within barcode groups. Due to differences in reads per sequencing run, different thresholds were used to screen sequences prior to analysis. For cross 1, reads were required to be present 3 or more times per barcode to be analyzed further. For cross 2, GAIIx reads were required to be present 7 or more times, while reads sequenced on the HiSeq were required to occur 10 or more times. Sequences were then compared between parental samples to identify differential markers—either by sequenced polymorphism or presence/absence [33], and differential markers were mapped to the reference *S. cerevisiae* genome using Bowtie version 0.12.7 [52]. Only reads which mapped uniquely were considered as markers. Genomic markers were selected to be at least 10 kilobases away from the next nearest marker. For cross 1, 452 markers were identified and used for QTL mapping. For cross 2, 441 markers were identified and used for QTL mapping. Marker positions, sequences, and average read number per marker can be found in Table S1. All marker positions provided refer to the beginning of the read when mapped to the reference genome. For cross 1, average read number for presence/absence markers was 60, for sequence polymorphism markers it was 66. For cross 2, average read number for presence/absence markers was 157, for sequence polymorphism markers it was 145.

Final genotyping data can be found in Tables S2 (cross 1) and S3 (cross 2). A genetic map for each cross was created using Mapmaker/EXP version 3.0 (Whitehead Institute, Cambridge, MA). WinQTL Cartographer version 2.5 [53] was used to map QTL via composite interval mapping (CIM) as described previously [22], [33]. Thresholds for significance were set using 1000 permutations of each dataset.

### Growth and Sporulation Measurement

Yeast were grown in standard Yeast-Peptone-Dextrose media (YPD, 1% yeast extract, 2% peptone, 2% dextrose). Hybridization during crossing was selected for by supplementing with G418 (200 mg/L, Invitrogen) and hygromycin (300 mg/L, Roche) and selecting for resistance to both drugs. Offspring tetrads were checked to confirm 2∶2 segregation for drug resistances. Gene knockout during reciprocal hemizygosity analysis was selected for by supplementing with nourseothricin (100 mg/G, Werner BioAgents).

The sporulation phenotype was assessed using flow cytometry to read out the SPS2::GFP marker as described previously [33]. Briefly, strains were grown at 400 rpm in 500 µL 96 well plate cultures in YPD for 15 hours, then 8 µL of the overnights were transferred to 400 µL 1% potassium acetate. After 30 hours, strains were frozen at −80°C prior to analysis by flow cytometry. Ideally, greater than 14,000 cell counts were used per offspring, per replicate. For cross 1, offspring were required to have at least 2000 counts for the trial to be recorded, and at least 2 trials for an average to be calculated. These limits were necessary as UCD51 and offspring have low cell viability during sporulation. Replicates were averaged to produce the final phenotyping data found in Tables S2 (cross 1) and S3 (cross 2). While the offspring phenotypes have a non-normal distribution, we have found that transformations do not normalize the phenotypes, affect the amount of epistasis we observe, or have an effect on QTL mapping [22], [30], so we performed our mapping analysis using untransformed phenotype values.

Broad sense heritability (H^2^) was calculated as described previously [30].

### Polymorphism Rate Analysis

QTL located on chromosomes 2, 4, and 15 in cross 1 and chromosome 7 (at 838 kb) in cross 2 were assessed as follows. We identified sporulation genes found in the 99% confidence interval (approximated as a 2-LOD drop from the peak apex) of each QTL and selected an equal number of non-sporulation control genes. Sporulation genes were defined as those which had reduced sporulation in a survey of the yeast deletion collection, while genes whose deletion did not affect sporulation we considered to be non-sporulation genes [16]. These criteria identified 27 sporulation genes: 9 on chromosome 2, 11 on chromosome 4, 5 on chromosome 7, and 2 on chromosome 15. An equal number of non-sporulation genes from each interval were selected as a control group. Coding region sequences for these genes were identified in assemblies from oak (YPS606) for all genes, UCD51 for genes in QTL found from cross 1 (chromosomes 2, 4, and 15) and M5 for genes on chromosome 7 (838 kb QTL). Selected genes, sequences, and polymorphism counts can be found in Table S4. In cases where our sequencing did not completely cover the coding region, gene fragments were aligned to the reference sequence before comparing oak and vineyard SNPs. We aligned oak and vineyard sequences using CLUSTAL W [54] to identify polymorphisms. Using the number of polymorphisms per aligned kb we performed a Wilcox test in R to determine if the two groups of genes had different polymorphism rates. Draft whole genome assemblies for UCD51 (BC106) and M5 (BC242) were deposited as Whole Genome Shotgun projects at DDBJ/EMBL/GenBank under the accession numbers JPXA00000000 and JPXB00000000.

### Reciprocal Hemizygosity Analysis

Reciprocal hemizygosity analysis of putative causal genes underlying QTL was performed as described previously [29], [33] with the following modifications. Genes to be tested were knocked out with the *natMX4* cassette, which confers resistance to nourseothricin [51]. For RIM15, 6 knockouts of the UCD51 allele and 5 knockouts of the YPS606 allele were used. For RIM101, 5 knockouts of the UCD51 allele, 6 knockouts of the YPS606 allele, and 6 knockouts of the M5 allele were used. In all cases, each hemizygous hybrid strain was phenotyped at least 5 times, and the results of these technical replicates were averaged.

### Causal SNP Identification

Identifying the causal change in *RIM101* was accomplished by random replacement of the BC593 (YPS606, ho-, ura3-) *RIM101* gene with the BC815 sequence as described previously for the identification of the *RSF1* causal polymorphism [22]. Random crossing-over among the 29 strains assayed created 10 subsets of potential changes across the gene. Phenotyping was performed without supplementing YPD with uracil (as was done in the case of RSF1) as supplementation caused all strains to sporulate above 90%, which made differentiating between polymorphism groups challenging. T-tests were performed on each subset region to determine if strains containing oak and vineyard alleles in that region were significantly different from each other.

Once we identified a region with a significant difference, we tested each SNP in that region for causality by replacing each oak allele with the vineyard allele. To create these single allele swaps, we again started with the ho-, ura3- YPS606 oak strain BC593 and replaced the region of *RIM101* containing all 4 alleles with the pCORE cassette [55], then used stitching PCR to create 4 replacement constructs, each replacing a single allele. Once each construct was integrated to replace pCORE, transformants were Sanger sequenced to confirm a single allele change. Two sequenced allele replacements for each position were backcrossed to the BC248 parent isolate. Additionally, two complete locus (coding and noncoding) replacements created during the initial random crossing over phase were backcrossed to BC248 to create a full locus swap strain. Multiple replicates of diploid, ura3+ progeny containing each vineyard allele were phenotyped for sporulation efficiency as described previously, except that sporulation was assayed at 8 hours rather than 30 hours. To control for timing variability during flow cytometry at such an early timepoint, each plate was frozen twice and run on the cytometer in both forward and reverse order, then these numbers were averaged.

For *RIM15*, only the polymorphism causing the P1271H substitution was tested. Again, the region containing the polymorphism was replaced using pCORE in BC593. Then pCORE was replaced with a construct containing the single nucleotide swap. This replacement was confirmed by Sanger sequencing. Two correct alleles were backcrossed to BC248, and replicates of diploid, ura3+ progeny containing the allele replacement were phenotyped as described for the *RIM101* swaps.

### *RIM101* and *RIM15* Allele Replacement Panels

Each causal SNP swap from above (*RIM101* G746T and *RIM15* C3812A) was crossed into our existing oak background allele replacement panel of 16 strains containing all combinations of the 4 polymorphisms previously identified [22]. Since the two polymorphisms in *IME1* are only 1521 bp apart and are unlikely to be separated by crossing over, three crosses were needed to obtain the complete panel. For each polymorphism, one *RIM101* or *RIM15* SNP replacement oak strain created above (each containing the *SPS2*::GFP fusion marked by the *hygMX4* cassette) was crossed to the (in order, *RME1*, *RSF1*, *IME1*-coding, *IME1*-noncoding) VVVV, VVOV, and VVVO strains (which contain the *SPS2*::GFP fusion marked by the *kanMX4* cassette) from the oak background allele replacement panel. Hybridization was selected for as described previously and resulting hybrids were sporulated and dissected to produce homozygous offspring.

Offspring were screened via RFLP to assemble two new panels of 16 oak stains containing all combinations of the oak and vineyard alleles of *RME1*, *RSF1*, *IME1*-NC, and *IME1*-C but only the vineyard allele of *RIM101* or *RIM15*. Each of these were combined with the original panel of 16 strains containing the oak alleles or *RIM101* and *RIM15* to create two new allele replacement panels, consisting of all combinations of *RIM101*, *RME1*, *RSF1*, *IME1*-NC, *IME1*-C (designated *RIM101* AR panel) and all combinations of *RIM15*, *RME1*, *RSF1*, *IME1*-NC, *IME1*-C (designated *RIM15* AR panel). Each panel was phenotyped 24 times for sporulation efficiency as described previously and the phenotypes were used to build backwards regression linear models using BIC to calculate coefficient significance in R to explore interactions among alleles. Percent variance explained was calculated using a sum of squares method on the residuals by dropping each coefficient independently and recalculating the model predictions without refitting the remaining coefficients.

## Supporting Information

Table S1 Marker locations, sequences, and average read counts. Cross 1 (first tab) and 2 (second tab) marker names, chromosomal locations, sequences, and average read numbers are listed. Location refers to the start of the mapped read when aligned to the reference genome. For sequence polymorphisms both oak and vineyard sequences are given, for presence/absence markers, one sequence is provided and the other is marked as “Absent Allele.”
(XLSX)

Table S2 Cross 1 genotypes and phenotypes. Each column is an offspring, each row is a marker. Phenotypes are located in the final row and are averages of multiple technical replicates. Genotypes are coded as follows: ‘A’ for a YPS606 Oak marker, ‘B’ for a UCD51 Vineyard marker, and ‘-’ indicates a missing genotype.
(TXT)

Table S3 Cross 2 genotypes and phenotypes. Each column is an offspring, each row is a marker. Phenotypes are located in the final row and are averages of multiple technical replicates. Genotypes are coded as follows: ‘A’ for a YPS606 Oak marker, ‘B’ for an M5 Vineyard marker, and ‘-’ indicates a missing genotype.
(TXT)

Table S4 Secondary QTL SNP survey. Table contains reference, oak, and vineyard sequences and polymorphism counts for 27 candidate sporulation genes and an equal number of controls, drawn from the 99% confidence intervals for the QTL on chromosomes 2, 4, 7, and 15.
(XLSX)
